# Oyster toadfish (*Opsanus tau*) boatwhistle call detection and patterns within a large-scale oyster restoration site

**DOI:** 10.1371/journal.pone.0182757

**Published:** 2017-08-08

**Authors:** Shannon W. Ricci, DelWayne R. Bohnenstiehl, David B. Eggleston, M. Lisa Kellogg, R. Patrick Lyon

**Affiliations:** 1 Department of Marine, Earth and Atmospheric Sciences, North Carolina State University, Raleigh, North Carolina, United States of America; 2 Center for Marine Science and Technology, North Carolina State University, Morehead City, North Carolina, United States of America; 3 Virginia Institute of Marine Science, College of William and Mary, Gloucester Point, Virginia, United States of America; Institute of Deep-sea Science and Engineering, Chinese Academy of Sciences, CHINA

## Abstract

During May 2015, passive acoustic recorders were deployed at eight subtidal oyster reefs within Harris Creek Oyster Sanctuary in Chesapeake Bay, Maryland USA. These sites were selected to represent both restored and unrestored habitats having a range of oyster densities. Throughout the survey, the soundscape within Harris Creek was dominated by the boatwhistle calls of the oyster toadfish, *Opsanus tau*. A novel, multi-kernel spectral correlation approach was developed to automatically detect these boatwhistle calls using their two lowest harmonic bands. The results provided quantitative information on how call rate and call frequency varied in space and time. Toadfish boatwhistle fundamental frequency ranged from 140 Hz to 260 Hz and was well correlated (r = 0.94) with changes in water temperature, with the fundamental frequency increasing by ~11 Hz for every 1°C increase in temperature. The boatwhistle call rate increased from just a few calls per minute at the start of monitoring on May 7^th^ to ~100 calls/min on May 10^th^ and remained elevated throughout the survey. As male toadfish are known to generate boatwhistles to attract mates, this rapid increase in call rate was interpreted to mark the onset of spring spawning behavior. Call rate was not modulated by water temperature, but showed a consistent diurnal pattern, with a sharp decrease in rate just before sunrise and a peak just after sunset. There was a significant difference in call rate between restored and unrestored reefs, with restored sites having nearly twice the call rate as unrestored sites. This work highlights the benefits of using automated detection techniques that provide quantitative information on species-specific call characteristics and patterns. This type of non-invasive acoustic monitoring provides long-term, semi-continuous information on animal behavior and abundance, and operates effectively in settings that are otherwise difficult to sample.

## Introduction

Evaluating ecosystem services provided by restored oyster reefs is crucial in determining restoration success. Sampling fish and benthic communities at subtidal oyster reefs can be labor-intensive, and low-visibility conditions or sampling gear restrictions can bias estimates of the animal assemblages using reefs as habitat. Passive acoustics is becoming more widely used in marine environments as a way to monitor these subtidal habitats. This approach assumes that the sounds produced by one or more species can be used to track their relative abundance and/or changes in behavior in space and time.

Many studies identifying fish calls have manually inspected spectrograms (time-frequency representation of the data) to assess the presence or intensity of call activity. While this approach may be effective, it is typically time consuming [1–4] and automated techniques are needed to process large volumes of data. In this study, a novel multi-kernel spectral correlation approach is used to identify toadfish boatwhistles. This pattern matching technique has some potential advantages over other automated techniques, such as traditional acoustic, band-limited energy detectors [5], in that 1) the presence of multiple harmonics and swept character of the signal may be considered in evaluating the detection, and 2) the fundamental frequency of each individual call can be readily returned along with its time and score.

Oyster toadfish (*Opsanus tau*) are among the most studied soniferous fish, and produce sound via rapid contraction of the muscles around the swim bladder [6–9]. Both male and female toadfish produce a suite of short duration pulse sounds, known as grunts, which are sometimes emitted in pulsed trains. These signals are often emitted as warning calls in agonistic situations [8, 10, 11]. The sound most associated with toadfish is the tonal “boatwhistle” call produced by males during spawning season as an advertisement to attract females to their nest site [6, 8, 12].

When toadfish move into shallow water from deeper-water overwintering sites in the spring for spawning, they become residents on oyster reefs [6, 13–15]. Like other resident reef species, oyster toadfish rely on the three-dimensional structure of oyster reefs for feeding, reproduction, and shelter from predators [14, 16]. Oyster toadfish feed primarily on benthic invertebrates, including small crabs and polychaetes [6, 14, 15]. The oyster toadfish is a known predator of xanthid crabs, including mud crabs (e.g. *Panopeus herbstii*), which prey on juvenile oysters [14, 15, 17, 18]. Studies examining the potential benefit of oyster toadfish have been mixed, with some finding a positive impact (i.e., toadfish decrease oyster mortality by modifying mud crab foraging behavior, [15, 19]) while others finding no impact (i.e., no impact on juvenile oyster survival or mud crab abundance, [17, 20]). Though the predator-prey relationships among toadfish—mud crab—oyster remain unclear, presence of toadfish can indicate suitable three-dimensional reef structure, which is beneficial to other economically and ecologically important reef species [14].

The Harris Creek Oyster Sanctuary in Chesapeake Bay, Maryland, USA provides an excellent opportunity to evaluate the utility of passive acoustics to monitor or assess restored oyster reefs. Researchers from the Virginia Institute of Marine Science designated eight reef sites within Harris Creek as part of an integrated assessment of oyster reef ecosystem services [21]. During May of 2015, a single hydrophone was deployed at each of these eight sites to investigate inter-reef soundscape differences. Inspection of the acoustic data revealed that the late-spring soundscape was dominated by boatwhistle calls of the oyster toadfish (*Opsanus tau*). A spectrogram correlation approach was used to detect individual boatwhistle sounds, elucidate environmental factors controlling the rate and harmonic frequency of calls, and explore differences in how toadfish utilize restored and unrestored reefs in Harris Creek.

## Methods

### Study location

**Fig 1:**
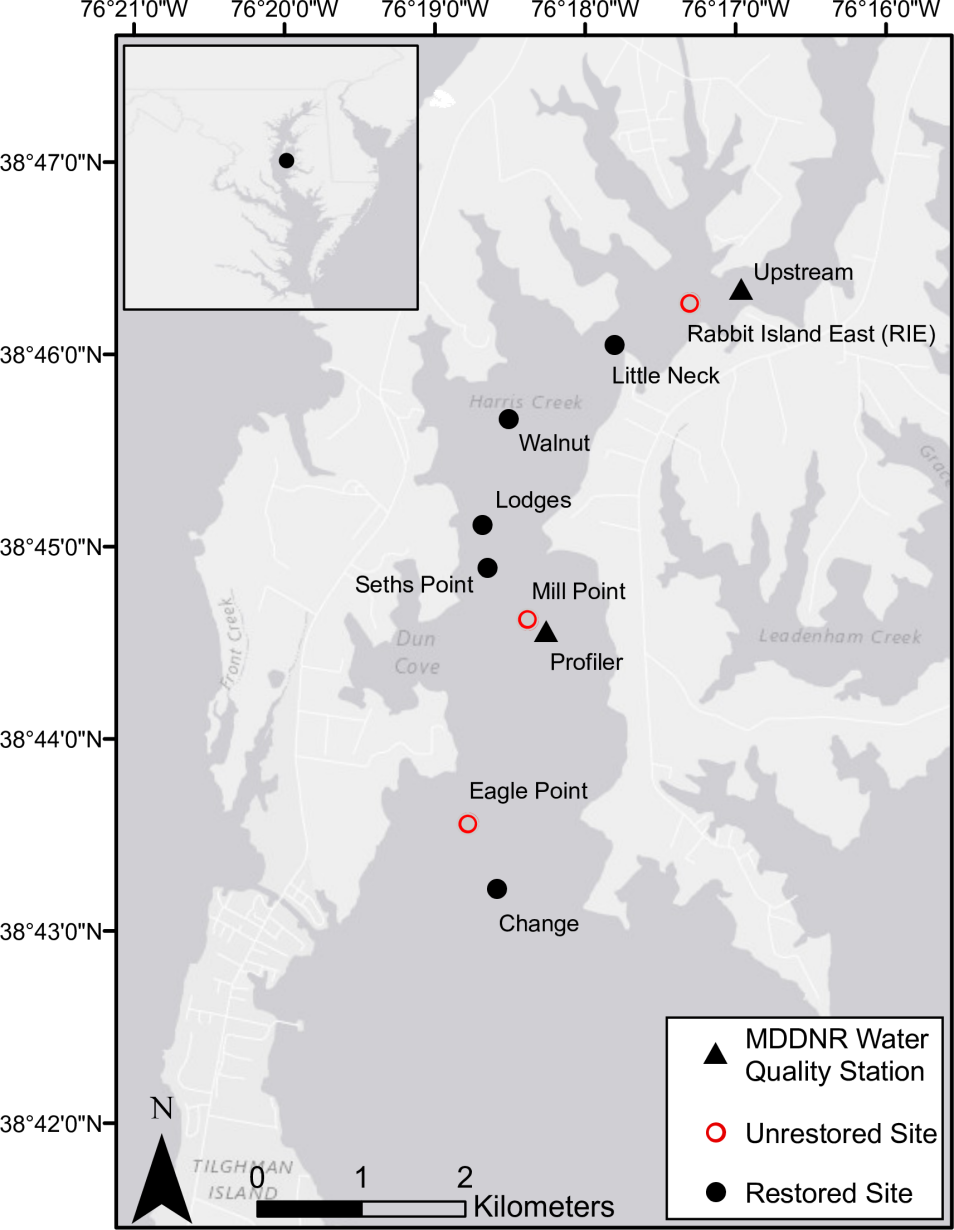
Map of Harris Creek study sites. Fig 1: Location of Harris Creek Oyster Sanctuary within Chesapeake Bay (inset) and map of study sites within the Harris Creek Oyster Sanctuary, Maryland. Red open circles are control, or unrestored sites. All other sites (black circles) were restored (spat-on-shell added) sites. Maryland DNR water quality monitoring stations are denoted by black triangles (Upstream Station XFG6431, Profiler Station XFG4618).

Harris Creek Oyster Sanctuary is the site of a large-scale oyster reef restoration effort. Recovery of the oyster populations is limited by both larval supply and availability of suitable settlement substrate. Initial restoration activities were completed in September 2015 and covered ~350 acres of bottom (Maryland Oyster Restoration Interagency Workgroup 2016). The eight sites utilized for this study are part of a broader effort to assess the ecosystem services provided by oyster reef restoration in Harris Creek (Fig 1). Three of the eight sites were control sites in which no restoration activities had occurred. The other five sites were reefs that were restored using juvenile oysters settled on oyster shell and planted directly on the substratum in 2012. All oyster larvae were produced and set on shell at the University of Maryland Center for Environmental Science’s Horn Point Laboratory. Oyster densities at each site were determined by the average number of live oysters in 1m^2^ tong grabs taken at each site in 2013 or 2014 surveys (Table 1). All work in Harris Creek was conducted under a scientific collection permit (SCP201514B) granted by the Maryland Department of Natural Resources. Although no vertebrate animals were collected or handled as part of the study described, companion studies did collect vertebrates and were covered by IACUC protocol.

**Table 1:** Oyster density (#/m^2^), tong sampling year, and restoration status of Harris Creek study sites.

	Oyster density (#/m^2^) +/- standard error	Sample year/number of grabs	Restoration status
**Rabbit Island East (RIE)**	13.6 ± 3.2	2014/16	Unrestored
**Little Neck**	128.1± 11.7	2013/96	Restored
**Walnut**	83.4 ± 13.2	2013/40	Restored
**Lodges**	86.4 ±12.9	2013/62	Restored
**Seth’s Point**	62.7 ± 9.3	2014/48	Restored
**Mill Point**	2.9 ± 0.8	2014/37	Unrestored
**Eagle Point**	17.9 ± 2.7	2014/30	Unrestored
**Change**	36.8 ± 7.1	2014/47	Restored

### Field recordings and data

Ambient sound was recorded simultaneously across the eight sites during a 26-day deployment in May 2015 using a set of SoundTrap underwater acoustic recording systems (Ocean Instruments New Zealand) deployed at each site. The SoundTraps were mounted vertically to a metal post that was anchored within the seabed. The instruments were positioned ~0.5 m above the seabed and approximately 1.0–3.5 m below mean lower low water (MLLW) at all sites. The recording schedule was set to capture two-minutes of acoustic data every 30 minutes at a sample rate of 96 kHz. The recording system’s frequency response is flat (±3 dB) over the 0.020 and 43 kHz band.

Water quality data were obtained from two Maryland Department of Natural Resources (MD-DNR) sites within Harris Creek. Measurements were taken in the lower water column at 15-minute intervals at a station upstream (ID: XFG2810) from the restoration area, and at 1-hr intervals from a water column profiler deployed near Mill Point (Station ID: XFG4618). During the period of the hydrophone deployments, temperature, salinity, dissolved-oxygen and pH were recorded continuously at both stations; however, for the profiler station, valid turbidity and chlorophyll data are only intermittently available. A high correlation between stations is observed for temperature (r = 0.98±0.01). More modest correlations are observed for salinity, dissolved-oxygen and pH; however, turbidity and chlorophyll are poorly correlated among sites (Table 2). The range of each parameter pair is similar between monitoring stations and water quality conditions were normal at the time of the hydrophone deployments (Table 2).

**Table 2:** Summary of water quality data from upstream and mill point profiler stations.

Parameter	Units	Inter-Quartile Range Upstream	Inter-Quartile Range Profiler	Corr. (r)
**Temperature**	°C	21.85–25.31	21.2–24.62	0.98±0.01
**Salinity**	ppt	11.81–12.40	11.56–11.76	0.64±0.01
**DO**	% Sat.	89.50–97.70	90.00–99.85	0.77±0.01
**pH**	-	7.56–7.72	7.63–7.74	0.64±0.02
**Turbidity**	NTU	2.80–4.00	1.95–3.00	-0.17±0.02
**Tot. Chyll.**	mg/L	1.00–2.20	0.80–1.70	0.12 ±0.02

### Toadfish boatwhistle detection

In this study, a spectrogram correlation method previously used for detecting cetacean sounds [22] is adapted to detect the characteristic toadfish boatwhistle call. The toadfish boatwhistle is similar to many cetacean calls in that the call is composed of narrow-band harmonics that are swept in frequency, though the frequency sweep in the boatwhistle is small (~3 Hz variation). Based on inspection of the boatwhistles calls detected within Harris Creek, as well as previous descriptions within the literature (e.g., [2, 3, 10]), a suite of detection kernels is constructed to capture the first two harmonics of these signals. These kernels are idealized spectrograms (time-frequency representations) of boatwhistle calls that vary in their fundamental frequency. The similarity between these kernel templates and a spectrogram of the data is used to identify boatwhistles and track their call frequency.

Spectrograms of the data are created for each 2-minute-duration field recording and a smoothed version of the mean spectrum is removed. Spectrogram estimation uses a Hanning window of 16384 points with 80% overlap, resulting in a time step *ΔT* = 32 msec. Each window is zero padded to give a frequency resolution of *Δf* = 2.93 Hz. The spectrogram matrix is then trimmed to retain a narrower band (85–620 Hz) of frequencies, and eliminate portions of the spectra outside of the range necessary for toadfish boatwhistle call detection.

Following Mellinger and Clark [22], kernels are constructed using a hat function, with a positive peak, corresponding to one of the swept harmonics, flanked at higher and lower frequencies by negative troughs. These negative regions aid in noise rejection [22]. The kernel value k at a given time and frequency (*t*, *f*) is specified by:

$$x_{1}=f-[f_{0}-\frac{t}{d}f_{swp}];$$ (Eq 1A)

$$x_{1}=f-[f_{0}-\frac{t}{d}f_{swp}];$$ (Eq 1B)

$$k(t,f)=\left(1-\frac{x_{1}^{2}}{\sigma^{2}}\right)e^{-(\frac{x_{1}^{2}}{2\sigma^{2}})}+\left(1-\frac{x_{2}^{2}}{\sigma^{2}}\right)e^{-(\frac{x_{2}^{2}}{2\sigma^{2}})};$$ (Eq 2)

where x_1_ and x_2_ are the distances from a point in spectrogram to the peak of the first and second harmonics at time *t*; *f*_*s*wp_ is the magnitude of the down-sweep in the first harmonic over duration (*d*), and σ is the call bandwidth measured in Hz.

To capture variability in call frequency, a set of 51 kernels are constructed with fundamental frequencies (f_o_) ranging between 137.7 and 284.2 Hz. These values are selected to match the frequency bins of the data spectrogram, incrementing by Δf between kernels. Each kernel has duration of 243 msec (7*ΔT), which is the approximate duration of the shortest boatwhistle calls observed. A slight down-sweep of 3 Hz is applied to the fundamental frequency and the call bandwidth is set to 10 Hz.

**Fig 2:**
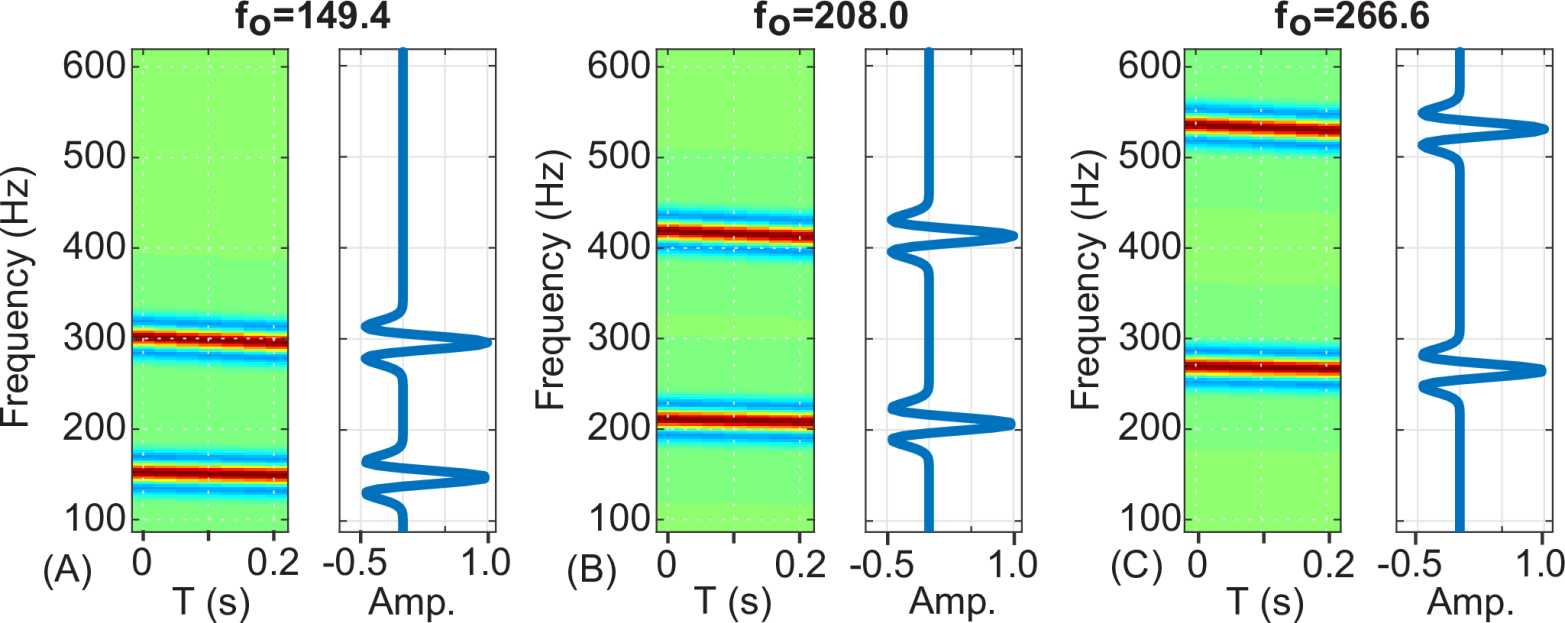
Example kernel spectrograms and frequency-amplitude profiles. Example spectrograms (left panels) and frequency-amplitude profiles (right panels) for three of the 51 kernels used. A) Kernel #5, f_0_ = 149.4 Hz; B) Kernel #25, f_0_ = 208.0 Hz; C) Kernel #45, f_0_ = 266.6 Hz. The detection process works by pattern matching (i.e. cross-correlating in the time dimension) the individual kernels against the data spectrogram.

**Fig 3:**
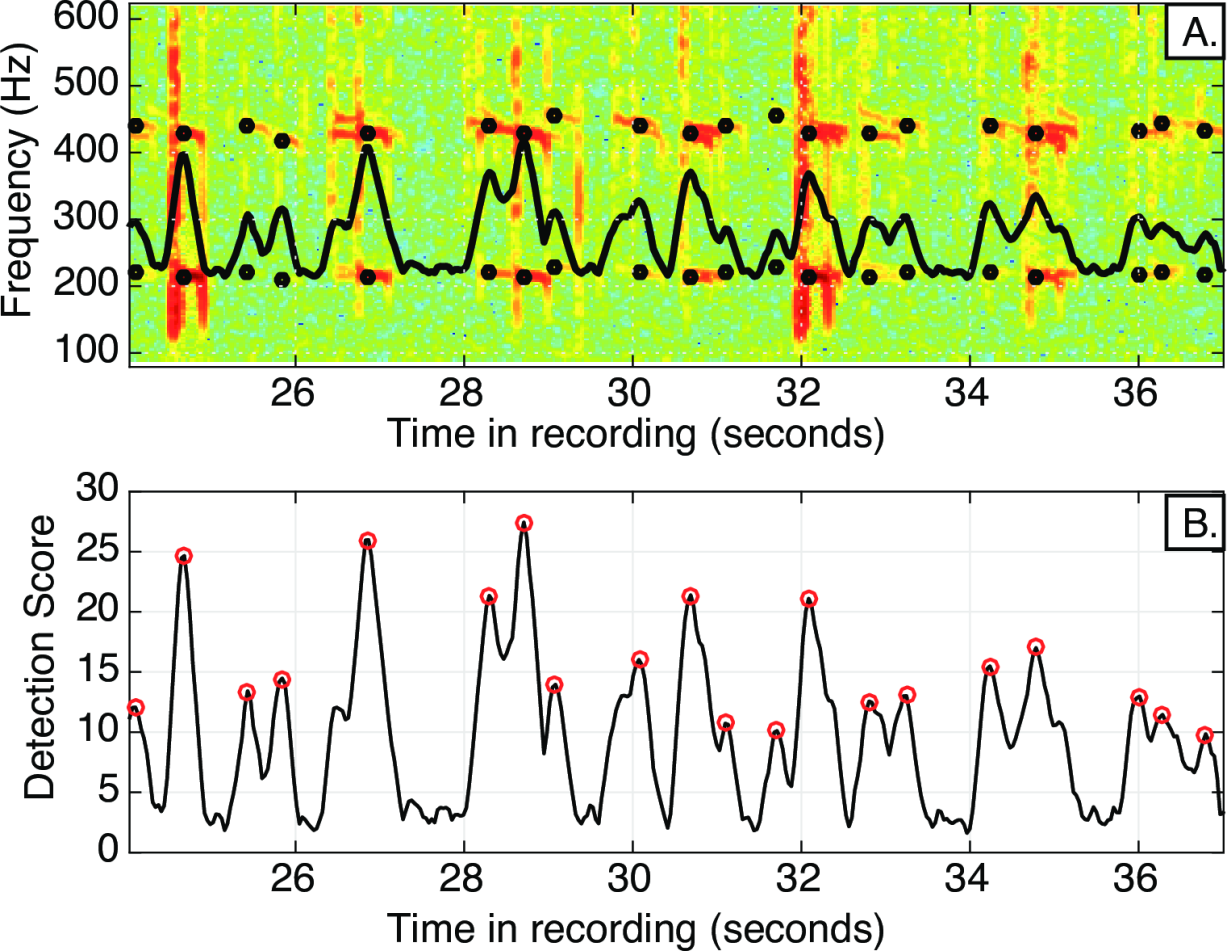
Example spectrogram and detector output. A). Spectrogram of a portion of a recording taken in Harris Creek in May 2015 shows detected toadfish boatwhistles. The black circles indicate the first and second harmonic detection frequencies. B) The detection score plot shows the associated detection score for each boatwhistle, with circles denoting the detection. The detection score represents the highest correlation value, returned from the suite of kernels, at each time step. The threshold for declaring a detection is determined empirically (See Methods: Detector sensitivity analysis).

The recognition score at each time step t is determined by cross correlating (in the time dimension) the data spectrogram with the suite of 51 kernels (Fig 2). The score at a given time is taken to be the maximum value returned from the suite of kernels. A peak detection algorithm is then applied to the recognition score time series and peak heights exceeding a threshold value are declared as detections. The time and score of the detection, along with the fundamental frequency of the best-correlated kernel are recorded (Fig 3).

### Detector sensitivity analysis

Because spectrogram levels are not normalized before the cross-correlation is computed, the maximum value of detection score is unconstrained [22]. The appropriate threshold to declare detection must be determined empirically. Ten recordings were randomly selected from each of the eight sites and the detector was run using an arbitrarily low threshold. An analyst reviewed these detections (N = 11200) and their associated data spectrograms, flagging any detection that could not be visually confirmed based on the time-frequency characteristics of the observed signal. The false detection rate was then calculated as a function of the score threshold. The results indicate a false detection rate of ~1% is obtained for a threshold score of 5.3 and this value is subsequently applied to all data, across all sites.

## Results

### Detection of toadfish boatwhistles

The detector returned a set of 1.2 million calls across the eight sites during the 26-day hydrophone deployment (Fig 3A). Some boatwhistles were associated with leading or trailing grunts, and many exhibited third and fourth harmonics within the data spectrograms. In some instances the amplitude of the first harmonic was less than that of the second harmonic, perhaps indicating a filtering effect due to propagation of the signals at shallow water depths [23]. Since the detector operated by matching only the lowest two harmonics, which were given equal weight in constructing the call kernels, these types of variability do not impact the sensitivity of the detector. The harmonic sound signatures associated with boat motor noise did occasionally trigger false detections; however, the patterns associated with toadfish calling were not influenced by these noise sources.

### Boatwhistle harmonic frequency

**Fig 4:**
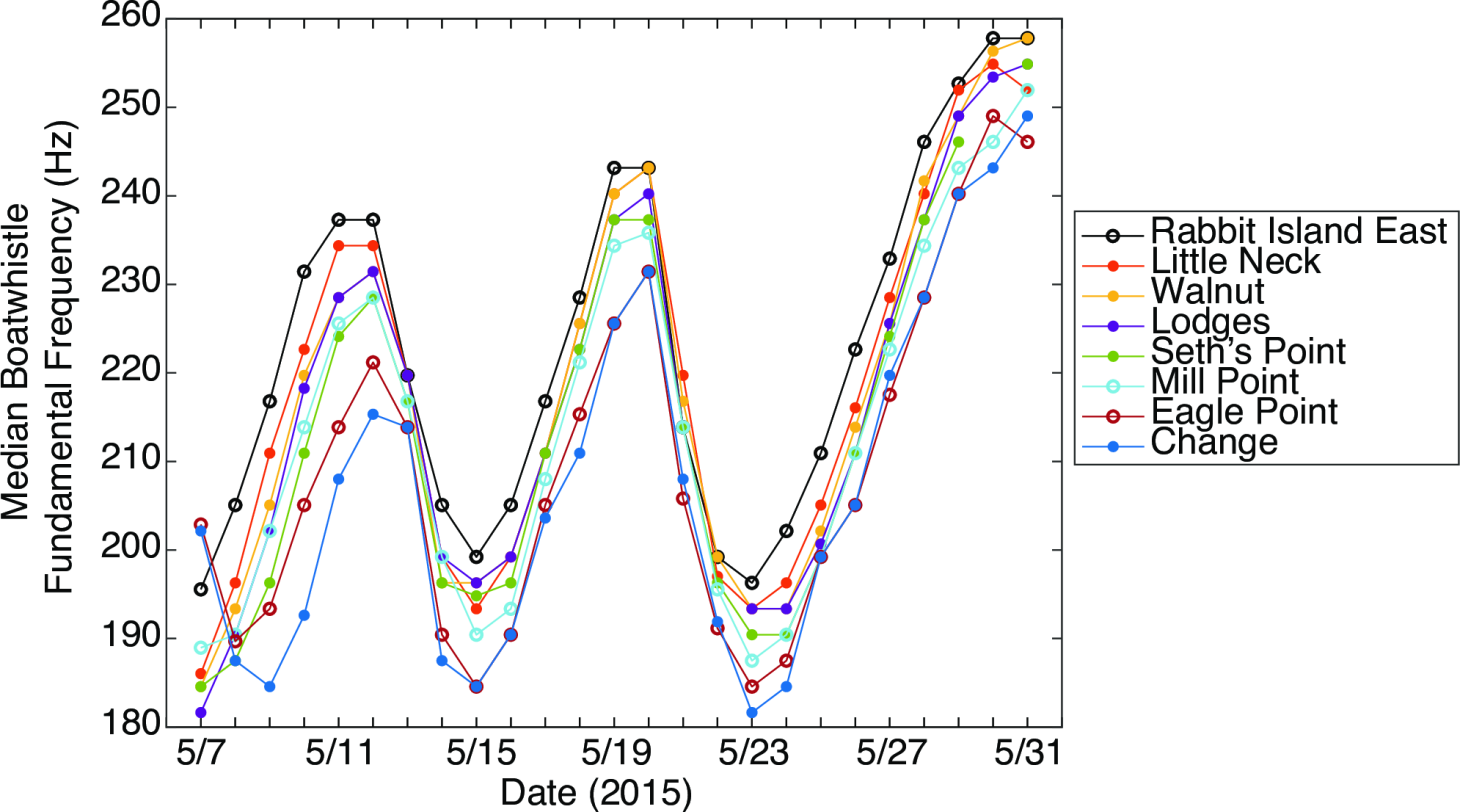
Boatwhistle fundamental frequency over time. Median fundamental frequency (Hz) of daily toadfish calls displayed for each of the eight sites along Harris Creek, MD. Open circles represent unrestored sites. Closed circles represent restored sites.

**Fig 5:**
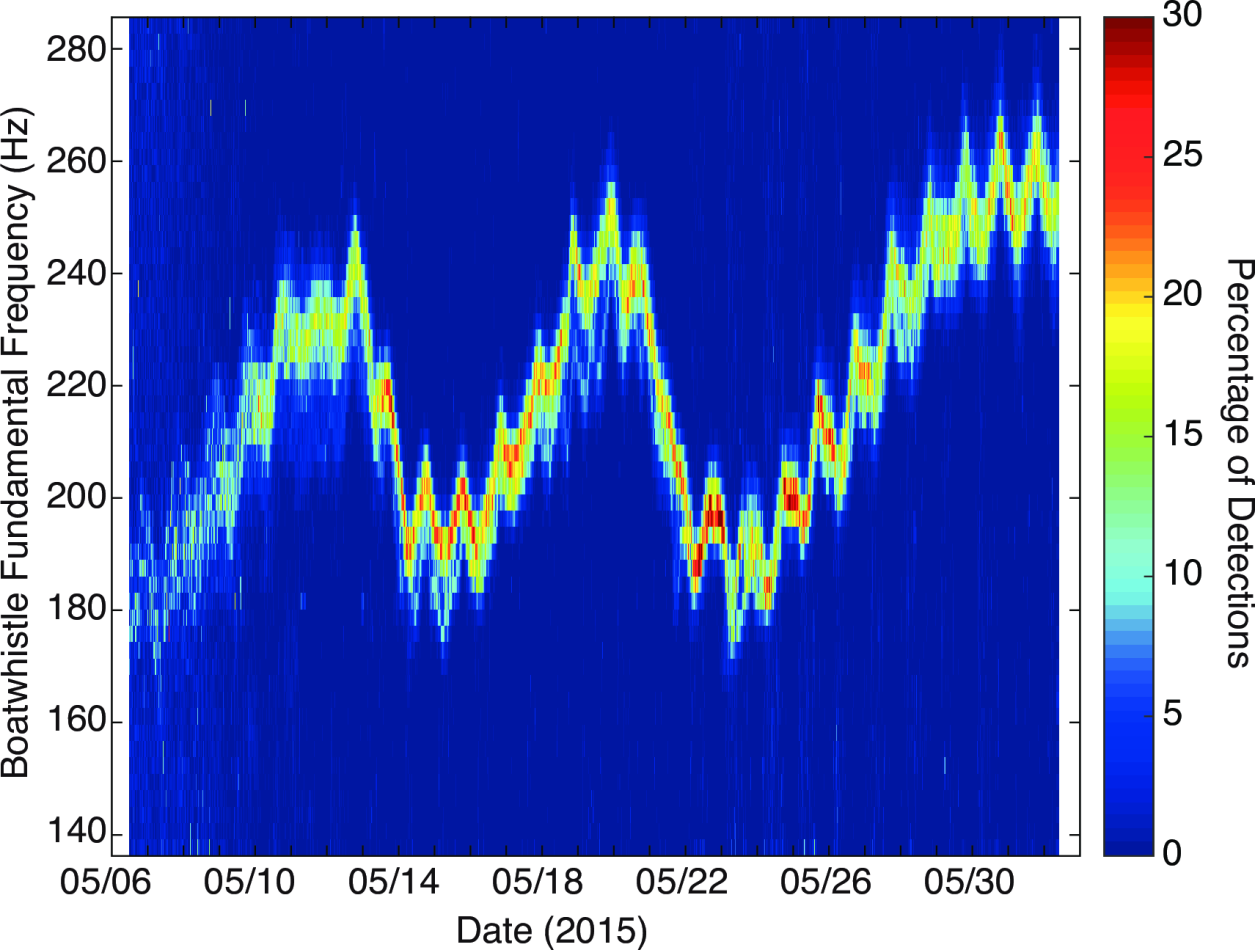
Data-density plot of toadfish boatwhistle fundamental frequency. Data-density plot showing fundamental frequency of toadfish boatwhistle detections (within each recording window) averaged across the eight sites in Harris Creek, MD.

**Fig 6:**
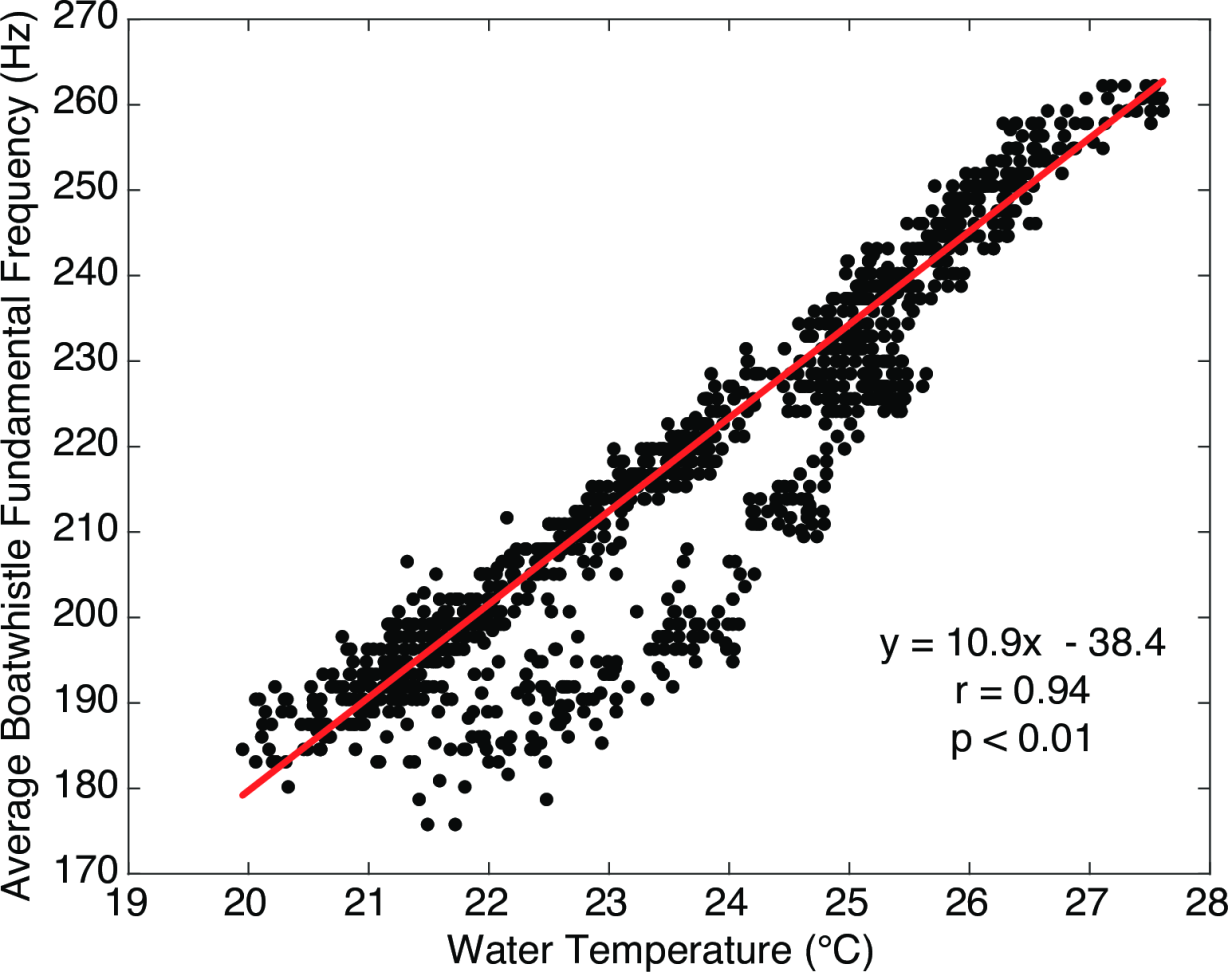
Boatwhistle fundamental frequency vs. temperature. Toadfish boatwhistle fundamental frequency (Hz) averaged across all eight sites versus upstream station water temperature (°C) recorded in Harris Creek, MD in May 2015.

**Fig 7:**
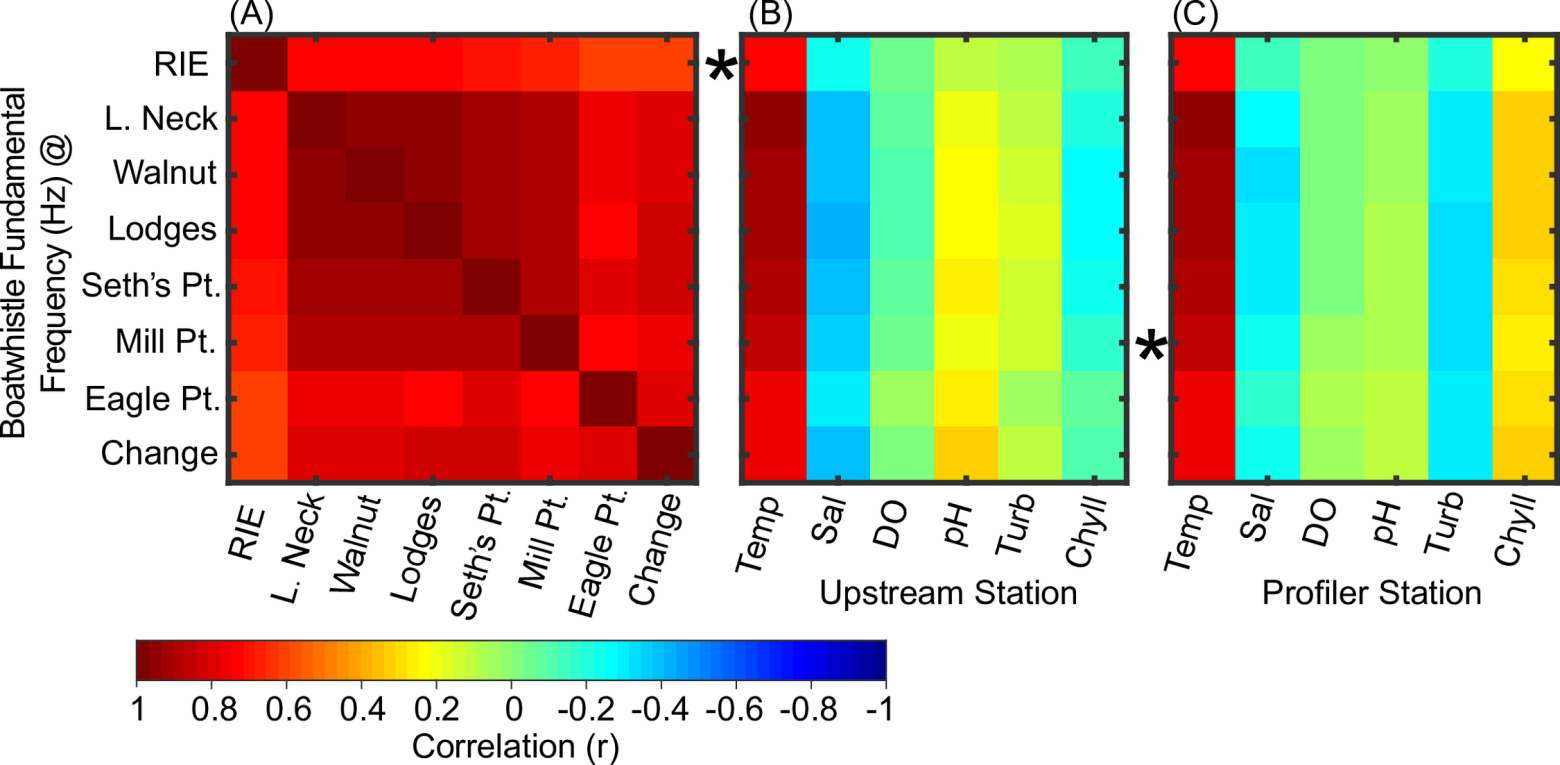
Correlation matrix for fundamental frequency and environmental variables. Correlations of boatwhistle call frequency (median value determined per recording) between hydrophone sites (A) and with regard to environmental time series recorded upstream of Rabbit Island East (B) and at the profiler station near Mill Point (C). Each row represents a hydrophone site (ordered by latitude, see Fig 1). The closest hydrophone site to each water quality monitoring station is marked with a star.

The harmonic frequency of the toadfish boatwhistles was consistent between sites: a majority (>60%) of calls detected at each recording time varied by no more than two spectral bins (*Δf* = 2.93 Hz) from the median across all sites (Figs 4 and 5). However, the fundamental frequency varied as a function of time, ranging from a minimum of 140 Hz to a maximum of 260 Hz. There was a strong (r = 0.94, p <0.001), positive relationship between water temperature and fundamental frequency (Fig 6). Other environmental variables were not strongly correlated with call frequency (Fig 7).

**Fig 8:**
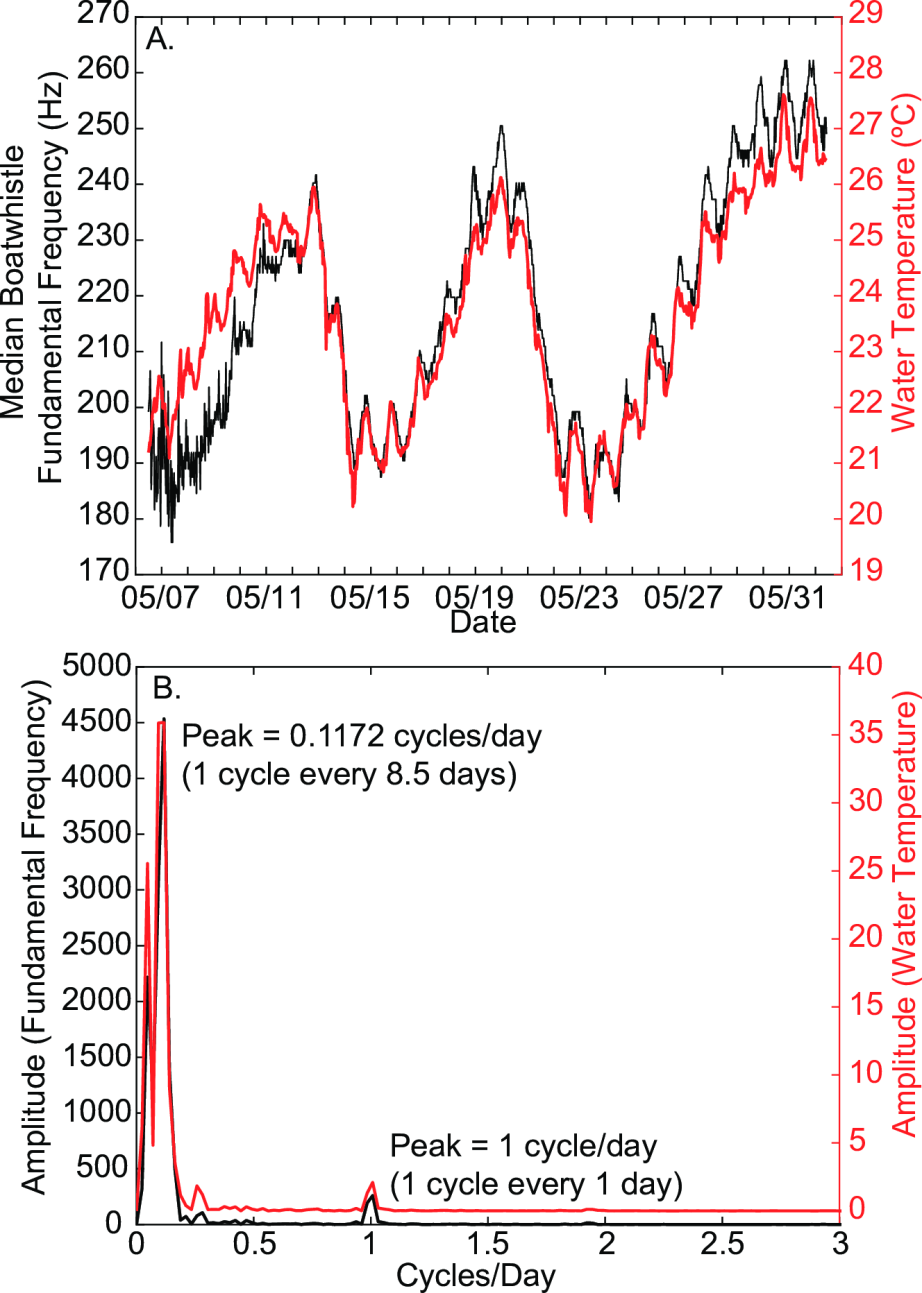
Periodicity of fundamental frequency and water temperature. A) Fundamental frequency of toadfish boatwhistles (black) and water temperature (red) versus time. Fundamental frequency is averaged within each recording window (30-minute intervals) across all 8 sites, and water temperatures are taken from the upstream monitoring station. B) Spectrum of the boatwhistle fundamental frequency and the water temperature time series. These datasets vary in phase with one another at periods of 1.0 and 8.5 days.

Further exploration of this periodicity in the harmonic frequency revealed two dominant periodicities, a once per day periodicity, and a significant and strong periodicity of every eight days (Fig 8). These patterns in harmonic frequencies match temporal patterns observed in water temperatures, which peaked once per day, and had another longer cycle of around 8.5 days (Fig 8). The 8.5-day period also matched the periodicity of local cross-shelf wind velocities, which are associated with the passage of weather systems.

### Boatwhistle call rates

**Fig 9:**
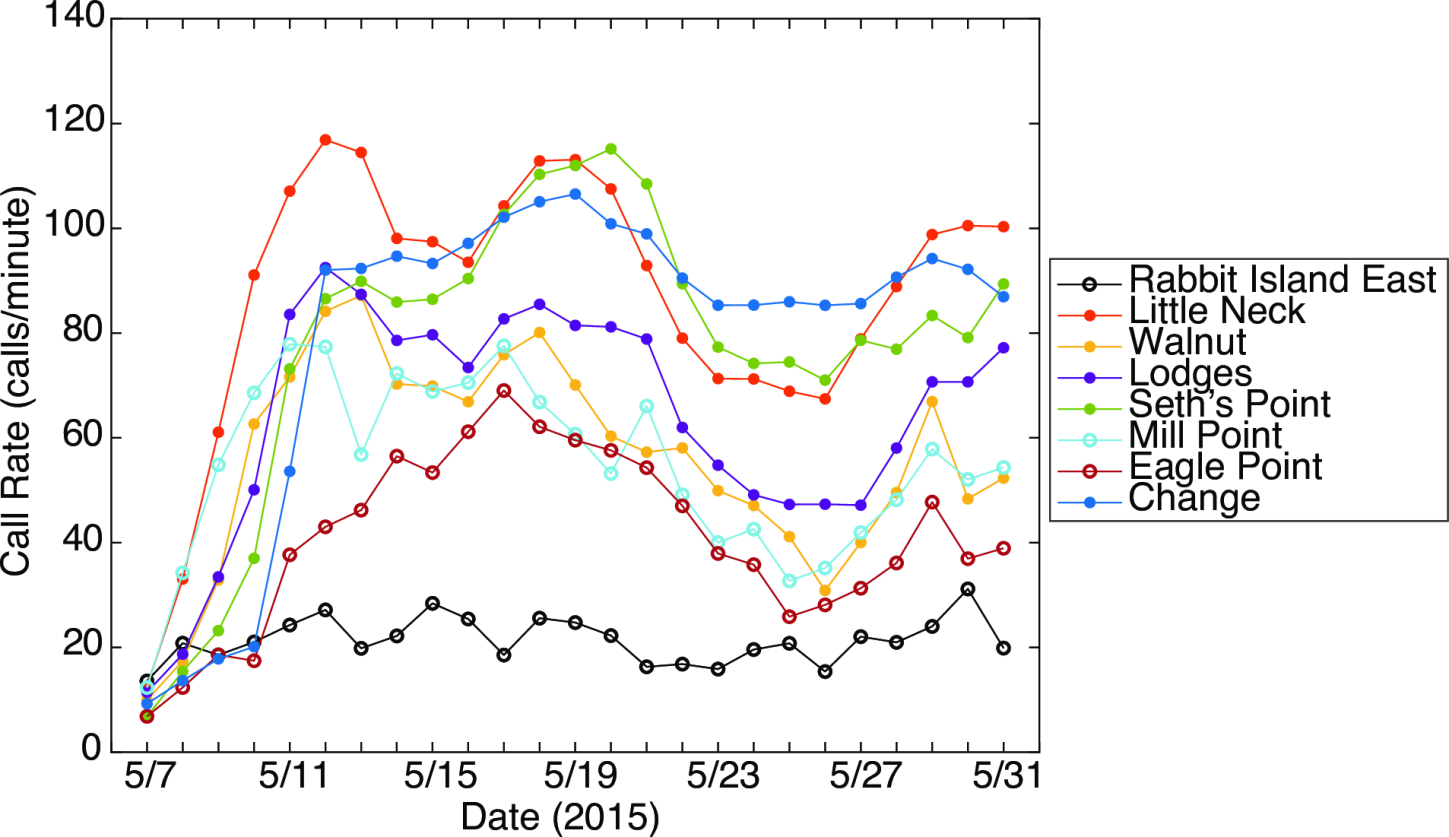
Boatwhistle call rate over time. Daily mean toadfish call rate (calls detected/minute) over time at eight sites in Harris Creek, MD. Open circles represent unrestored sites. Closed circles represent restored sites.

Toadfish call rate was low at all sites at the beginning of the deployment, and increased rapidly for nearly all sites within the first four days of recording (5/7/15 to 5/11/15) (Fig 9). Call rates were highest at Little Neck, Change, and Seth’s Point, averaging 80 to 110 calls per minute for most of the deployment (Fig 9). Call rates were lowest at Rabbit Island East, averaging less than 20 calls per minute during this recording period (Fig 9). Sites with higher call rates were restored sites with higher oyster densities (36.8–128.1 oysters/m^2^) whereas Rabbit Island East was an unrestored site with low oyster density (13.6 oysters/m^2^) (Table 1).

**Fig 10:**
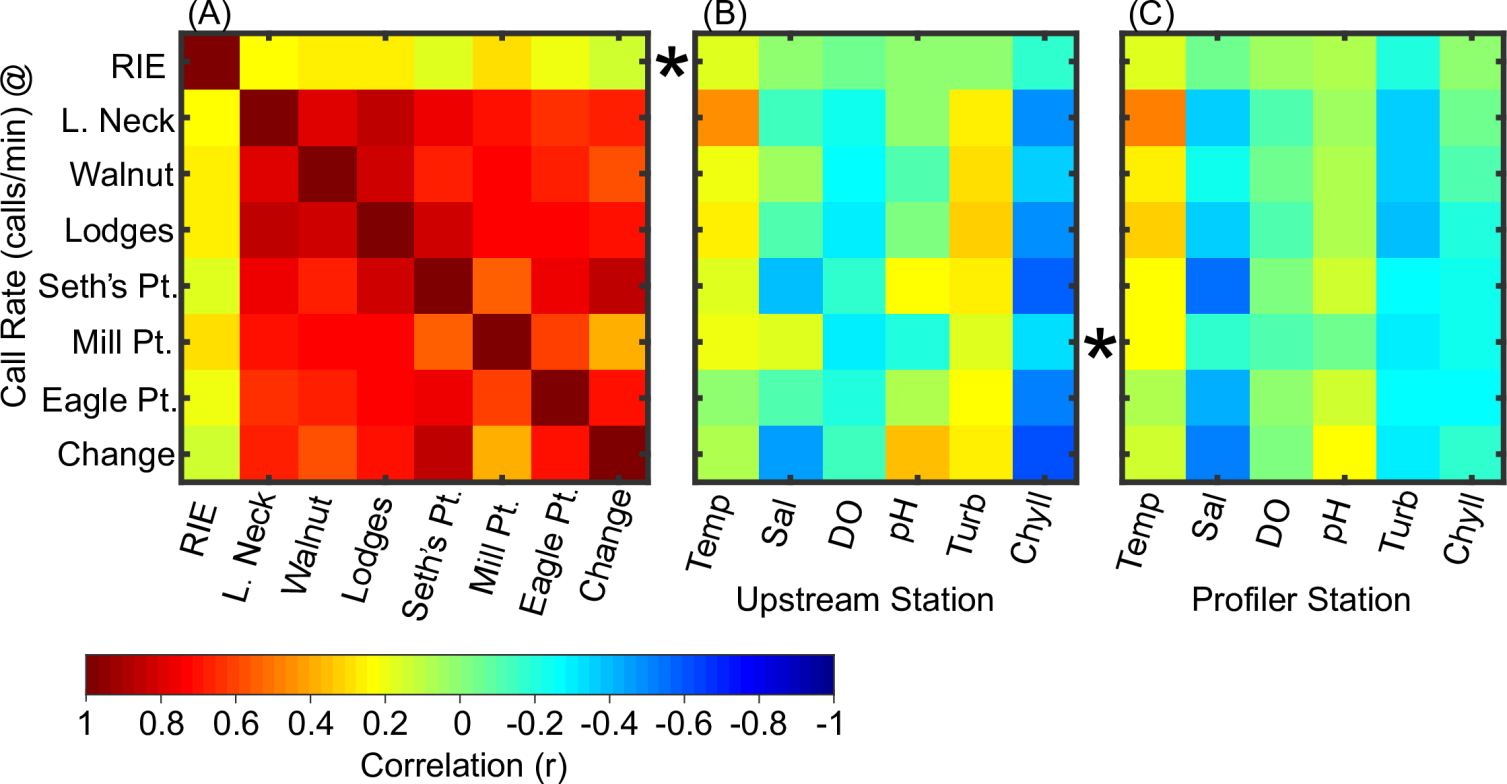
Correlation matrix for call rate and environmental variables. Correlations of boatwhistle call rate (estimated per recording) between hydrophone sites (A) and with regard to environmental time series recorded upstream of Rabbit Island East (B) and at the profiler station near Mill Point (C). Each row represents a hydrophone site (ordered by latitude, see Fig 1). The closest hydrophone site to each water quality monitoring station is marked with a star.

**Fig 11:**
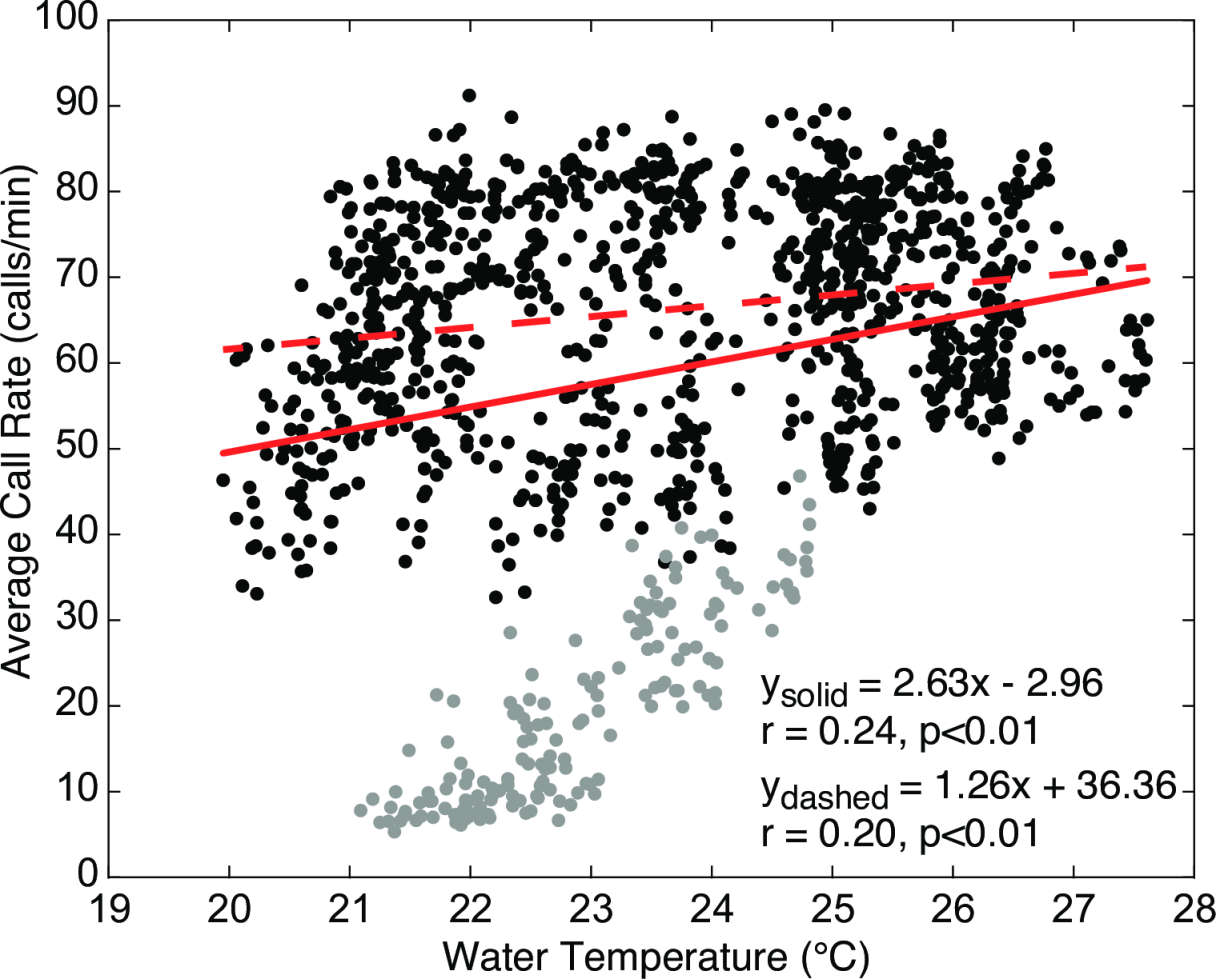
Call rate vs. water temperature. Mean boatwhistle call rate (calls/min) averaged across all sites and associated water temperature recorded from the upstream water quality station in Harris Creek, MD May 2015. Gray points denote call rate measurements during the first four days of monitoring, when the call rate is low or rapidly changing prior to the period of more active spawning and the water temperature also happens to be low due to its cyclic nature. Regression lines are given for models that include (solid) and exclude (dashed) data from this early period.

Although temperature and call fundamental frequency were strongly correlated, temperature does not explain much of the variation in call rate (r = 0.24, p<0.01) (Figs 10 and 11). No other environmental variables were strongly correlated with call rate (Fig 10).

**Fig 12:**
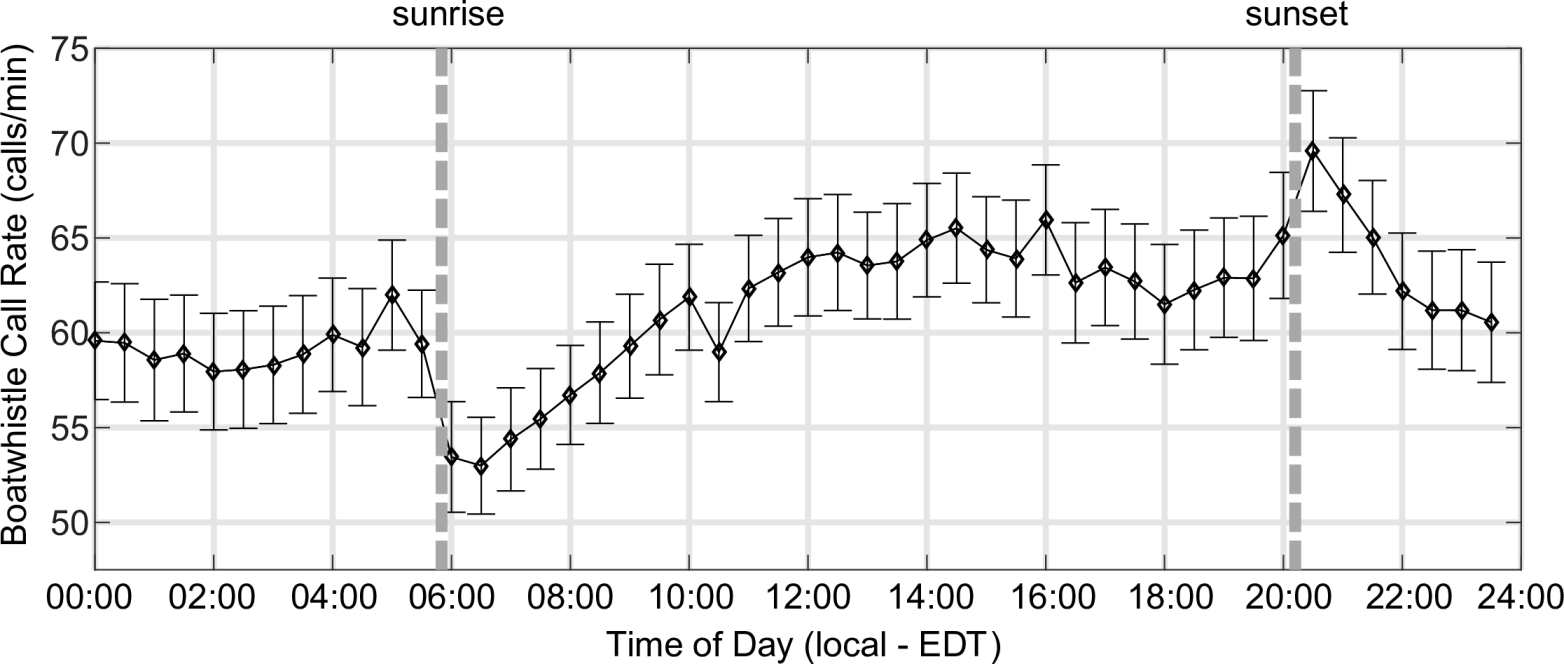
Boatwhistle call rate vs. time of day. Dashed vertical lines show local sunrise and sunset times in mid-May 2015. Vertical error bars show uncertainties in mean call rate (standard error) across the Harris Creek sites and are based on a jackknife resampling by site (i.e., files from each site are systematically excluded from the calculation of the mean rate at each sampling time).

Call rate varied with time of day, with call rates peaking just after sunset and dropping off drastically prior to sunrise (Fig 12). Following the drop in call rate at sunrise, call rates steadily increased during the morning hours, and then reached a plateau at an average of 65 calls/min during the day.

### Spatial patterns in boatwhistle call rates

**Fig 13:**
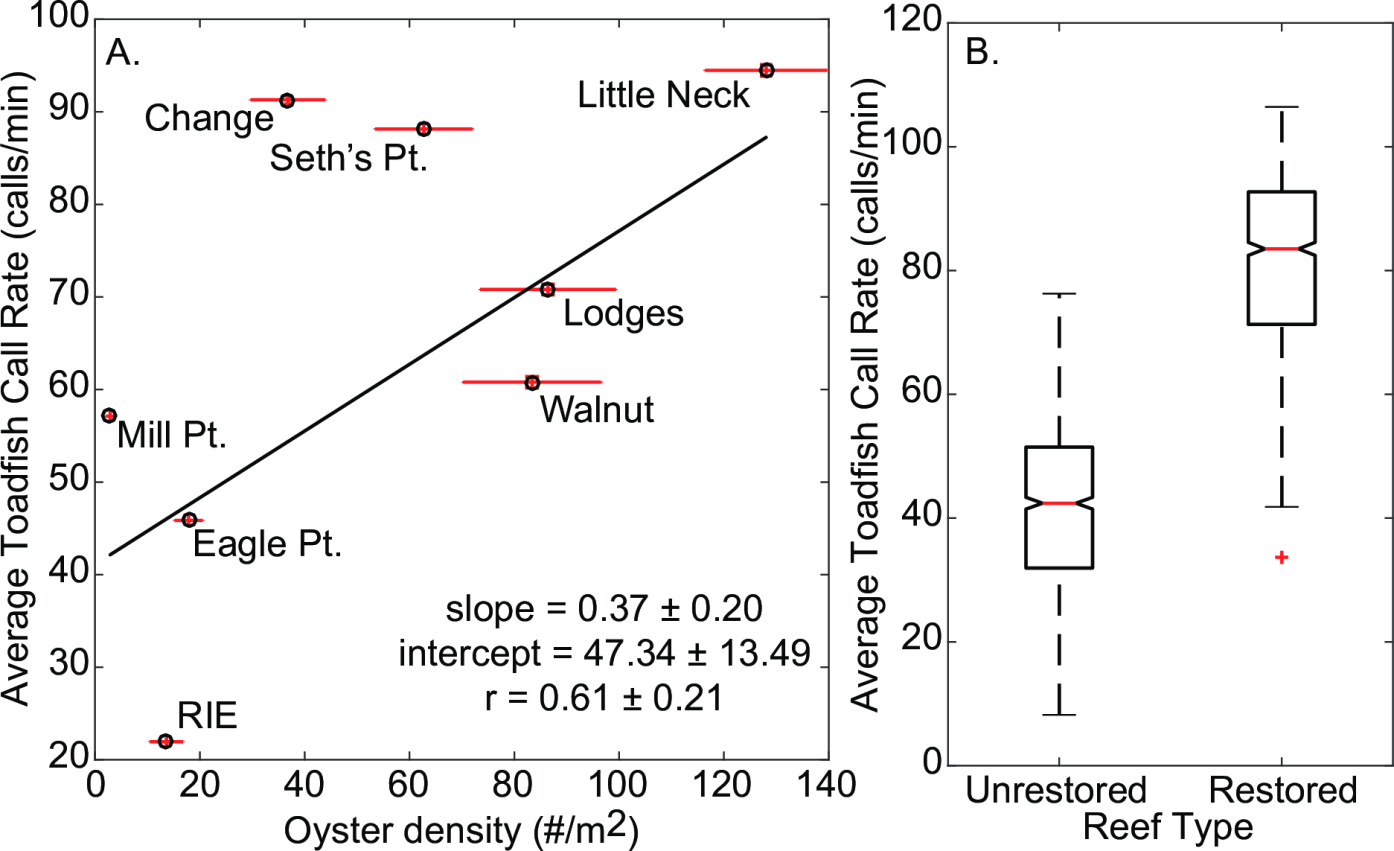
Call rate vs. oyster density and restoration status. A) Oyster density at each cultch reef site (number/m^2^) versus average toadfish boatwhistle call rate (calls/min) from reef sites in Harris Creek Maryland. Error bars are the standard error of oyster density (x) and average call rate (y). B) Average toadfish call rate (calls/min) from each reef type in Harris Creek, MD. Control reefs (n = 3 sites) had no restoration activities whereas treatment sites (n = 5 sites) were restored in 2012 with juvenile oysters set on shell. Only call rates after 5/11/15 were used in these calculations due to rapid changes in call patterns as a result of the onset of spawning.

There was a slight positive relationship between oyster density (#/m^2^) at each recording site and average call rate (Fig 13A). There was a significant difference between mean call rate between restored oyster reefs and control sites (Fig 13B; Kruskal-Wallis test, p<0.001). Mean call rate at restored oyster reefs was nearly double call rates at unrestored sites.

## Discussion and conclusions

### Detection of toadfish boatwhistles

As soundscape ecologists are now routinely collecting large volumes of passive acoustic data, automated detection techniques are becoming increasingly important to these efforts. A multi-kernel, spectrogram correlation approach provides an effective technique to identify and determine the harmonic frequencies of individual toadfish boatwhistle calls. While matched-filtering approaches such as these have not been widely applied to study fish vocalizations, they incorporate information on the time-frequency content of a signal that is not typically considered by band-limited energy detection techniques.

### Boatwhistle harmonic frequency

**Fig 14:**
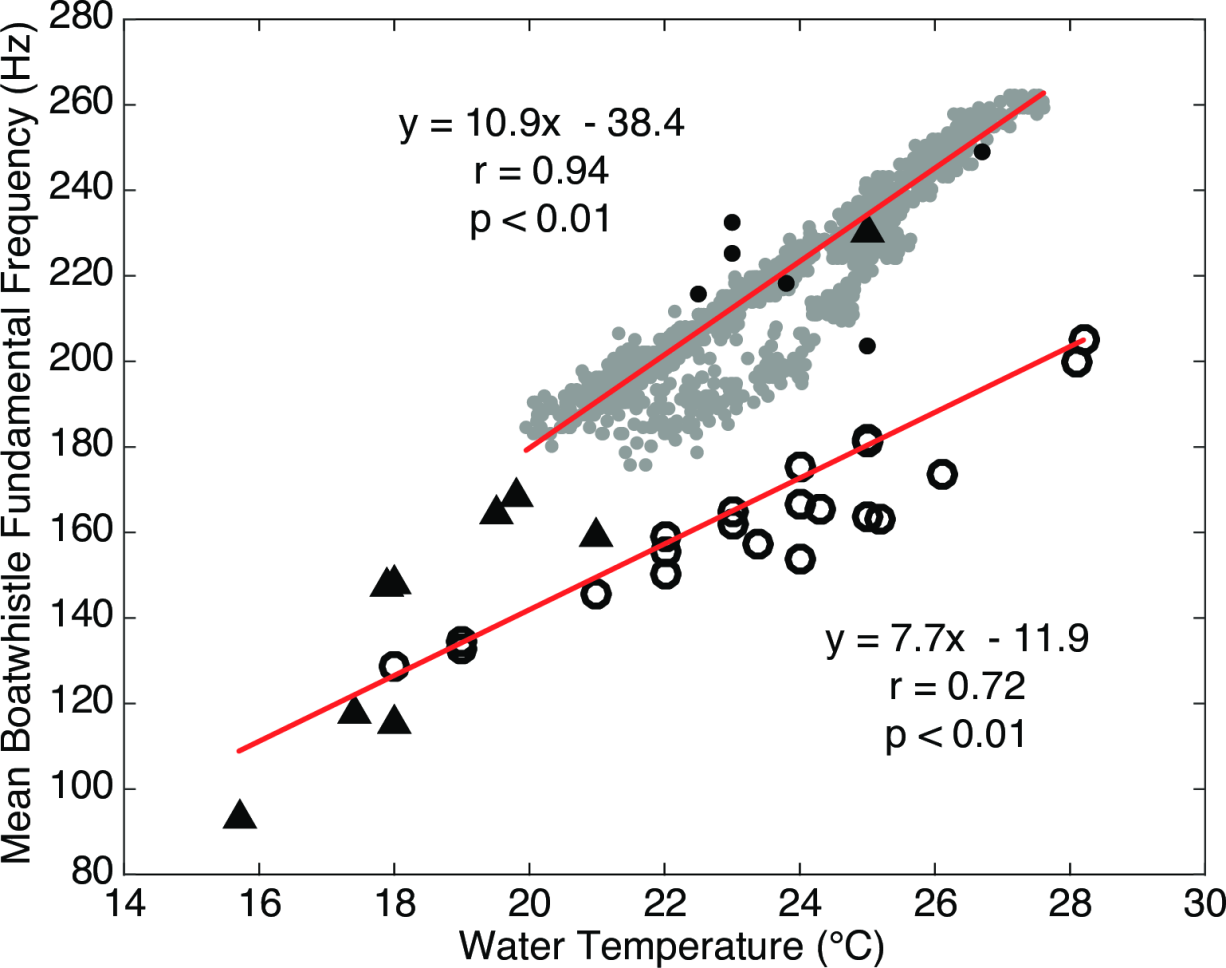
Historical comparison of boatwhistle fundamental frequency vs. water temperature. Boatwhistle fundamental frequency averaged across all sites and associated water temperatures for present day (2015, gray filled circles) recordings from Harris Creek, MD and from previous 1968 recordings in Delaware and Virginia during early season (16 May to 5 June 1968, black triangles), peak season (14 June to 15 July 1968, black filled circles), and post season (22 July to 23 October 1968, open circles) Toadfish boatwhistle characteristics from 1968 are from Tables 1 & 2 of Fine 1978.

The dependence of toadfish call frequency on water temperature was described early on by Tavolga [10]. Seasonal patterns of call frequency were later quantified by Fine [24] using data recorded weekly from sites in Virginia and Delaware collected during the period between May and October in 1968 (N = 45) (Fig 14). When plotted with present day call frequencies and water temperatures, the relationships are similar between the two data sets; however, the present day dataset indicates a greater change in fundamental frequency over the same increase in temperature (Fig 14). While the Harris Creek study recorded data only during the month of May, the fundamental frequency was assessed over much shorter interval of 30 minutes (N = 1248). This reveals that the frequency of the toadfish boatwhistle responds linearly to changes in water temperature at much shorter time scales.

Regression of the water temperature vs. fundamental frequency data from Harris Creek shows that call frequency increases by ~11 Hz/°C (Fig 14). Regression of all the data reported by Fine [24] yield a somewhat lower slope, ~8 Hz/°C; however, Fine [24] suggests that the relationship varies through the mating season with a decrease in slope after the peak of the mating season. Those data points from Fine [24] that are identified as being collected during the early-to-peak portions of the mating season, agree well with the trend of the data observed in Harris Creek.

Due to variation in toadfish boatwhistle call frequency observed over a relatively short time period, as well as longer-term variation observed in previous studies [24], it is important to consider this variation when developing effective detectors for use in multiple study systems.

### Boatwhistle call rates

Call rates increased rapidly from ~ 10 calls/min to as much as 120 calls/min within the first six days of the recording deployment. Though toadfish are considered reef resident species, previous tagging studies in the Chesapeake Bay revealed that this species has bi-seasonal movements associated with spawning [13]. After overwintering in muddy bottoms offshore, toadfish move into shallower water and find suitable habitat, often oyster reefs, to establish nests for spawning [6, 13, 25]. The dramatic increase in call rate is interpreted to indicate the beginning of the first spawning period where we have individuals moving into reef habitats from offshore wintering habitats. Spawning in early to mid-May is consistent with previous reports of toadfish spawning in Chesapeake Bay [13, 25].

Though environmental variables were not correlated with toadfish call rate, we did observe daily patterns in call rates, with call rate dropping significantly just before sunrise. This pattern was also observed in passive acoustic recordings off the West Florida Shelf and in the Florida Keys, with toadfish calling decreasing in the early morning (06:00h to 09:00h) and peaking around dusk [1, 2]. It is worth noting that unlike most soniferous fish that produce sound associated with spawning exclusively at night, toadfish in the current study, as well as previous studies [26], call throughout the day and night.

### Spatial patterns in boatwhistle call rates

A future goal of passive acoustic monitoring is to be able to relate soundscape characteristics to seascape characteristics in an effort to monitor habitats (e.g., [27–28]). Although there was only a weak positive relationship between call rate and oyster density across the eight sites, when grouped by restoration status, there is a significant difference in call rates between restored (spat on shell added) and unrestored reefs—with restored reefs having nearly twice as many calls as unrestored reefs.

An overarching goal of these Integrated Assessment Sites within the Harris Creek Oyster Sanctuary is to investigate ecosystem services of restored oyster reefs [21]. Although a study looking at fish and crustacean utilization of these oyster reefs found no difference between restored and unrestored reefs, the target species were more transient and may not adequately reflect the reef-associated community [21]. Given that tremendous effort goes into sampling the fish and benthic community at these subtidal sites, and that there is a detectable difference between the soundscape at restored vs. unrestored sites, can toadfish call patterns be used as an indicator of restoration success?

The oyster toadfish, like other oyster reef-associated fish, use hard substrate microhabitats, which oyster reefs provide, as nesting sites [6, 16]. Thus, increased call rates may indicate that there is sufficient structure to host reef resident species and provide necessary habitat space that serves as refuge from predators and as nesting sites. In addition, presence of oyster toadfish may also point to presence of other reef-associated species, including smaller fish like the naked goby, which are an important food source for the more transient, commercially important species that frequent oyster reefs [14].
